# Introducing *npj Biofilms and Microbiomes*

**DOI:** 10.1038/npjbiofilms.2015.4

**Published:** 2015-03-25

**Authors:** Staffan Normark

**Affiliations:** 1 Editor-in-Chief Department of Microbiology, Tumor and Cell Biology, Karolinska Institutet, SE-171 77 Stockholm, Sweden

Planet Earth is by far a microbial world, and microbial contribution is evidenced by the significant biomass and activities displayed by microbial biofilm communities in all habitats.

*npj Biofilms and Microbiomes* will feature new and exciting findings on biofilms and microbial communities: how member organisms interact at various levels; how they influence their respective environments; and the effects of external perturbations on structure function relationships.

Modern biology developed through studying relatively simple model systems, and major findings on key traits such as those of environmental stress responses were accomplished using mono species cultures of common laboratory bacteria. The full extent to which unicellular organisms exhibit multicellular behaviour, initially for populations and more recently for communities, was not realised until several decades later. For example, we now understand that microorganisms use various extracellular signalling mechanisms to establish communities where their collective activities are substantially different from the summation of the activities of individual members. Moreover, it was recently demonstrated that communication mechanisms using electron shuttling are employed by several bacterial species. Individual members of a population, however, can evolve new variants as a response to stress, which may impart benefits for the entire population. The emerging concept of bacteria as social organisms is a disciplinary milestone and reflects the convergence of microbiology and related life sciences, as well as ecology and microbial ecology. In addition, understanding the mechanisms of microbial population and community behaviour, using quantitative systems analysis and real-time imaging technologies, sheds light on complex social–microbial interactions and contributes to both ecological theory and experimental ecology.

The largely hidden powers of microbial biofilms hold much promise for applications across a broad scope of systems. These include the control of disease-causing microorganisms and the detection and management of pathogens in the environment; engineering microbial biofilms for effective bioremediation of pollutants; and the discovery of biofilm-generated novel bioactives. While the realisation of several biofilm applications is imminent, others may be reality only after robust tools for reproducible mixed-species microbial biofilm community analyses have been developed. Notably, to realise these opportunities, cross-disciplinary collaboration and systems need to be embraced, ranging from hydrodynamics at microscales to computational systems biology at the ecosystem level.

Advancing the fields of microbial community and biofilm biology calls for a change in how microbiology is perceived, practiced and studied. Biofilms are not just accidental mixtures of different types of microorganisms, rather their highly coordinated three dimensional architecture is built to accommodate unique and specific communal interactions. These interactions are often diffusion-limited and dictated by the relative spatial distribution of member organisms. Moreover, the polymeric matrix in which biofilm cells are embedded is a remarkably complex and active component of all biofilms, but remains relatively poorly understood. This matrix holds together microbial populations and communities in polymeric structural scaffolds that contribute to unique biofilm properties, such as stress resilience and long-distance communication mediated by directed electron shuttling. An array of such specific traits and properties is predicted to emerge from the increased efforts of contemporary biofilm research into this integral biofilm component.

The microbiomes of biofilm communities are now rapidly becoming focal points in studies of all ecosystems, from environmental and industrial to medical, driven by the notion that the well-being of these systems depends on the unique contribution of their microbiomes. In fact, microbial biofilms are the oldest known biological communities, dating back more than 3.6×10^9^ years. They have since evolved along with, and closely associated to, the more recent evolution of higher organisms, establishing a strong interdependence on a single holobiont. We can now explore the biology of host multicellular organisms, as well as the ecology of various habitats, by addressing their interactions within their respective microbiomes.

Technical advancements such as metagenomic, metatranscriptomic, metaproteomic and metabolomic approaches have provided tools to dissect virtually any natural polymicrobial community and assess responses to a multitude of external and internal perturbations. Many of these communities are highly complex and require sophisticated experimental design, data collection and analysis to attain meaningful results. The analysis of complex interactions in multispecies microbial communities will require increased efforts in computational biology, bioinformatics and overall systems biology approaches. *npj Biofilms and Microbiomes* will welcome manuscripts dealing with large-scale analyses of microbial biofilms and communities in various natural or engineered settings, but will prioritise those that provide mechanistic insights, and/or means to make use of or perturb the communities in ways that can be beneficial.

The human microbiome is a system that has recently attracted significant attention. The resulting wealth of data has established important correlations between the host’s various organs and the associated microbiomes. For example, the gut microbiome composition has been linked to various host conditions such as diabetes, atherosclerosis and autoimmune disease. Indeed, studies are now emerging on the effect of microbial fermentation products from dietary sources on intestinal gluconeogenesis, which subsequently affects the host’s glucose and energy homeostasis, and negative correlations between gut microbe gene richness and the likelihood of acquiring an autoimmune disease. Similarly, differential effects of vaginal versus caesarian birth delivery on shaping the infant’s initial gut microbes have been observed, a finding that is likely to result in directed manipulation of intestinal microbiome. Already today, patients suffering from *Clostridium difficile* infections are treated with particular complex microbial communities from healthy individuals, with promising outcomes. We can expect to see how such interventions will improve with further understanding of the various roles microorganisms have in healthy gut physiology. We are witnessing other exciting developments such as possible links between gut microbial activities and cognitive functions, as well as studies linking gut-derived microbial products and the shaping of the immune system.

Beyond the various human microbiomes, we know very little about the myriads of interactions between environmental microbial communities and a spectrum of hosts and surfaces in all ecosystems.

Future challenges will also involve a clearer explanation of the emerging host–microbiome interactions in mechanistic terms. *npj Biofilms and Microbiomes* very much wishes to become the journal for such contributions.

As the first Editor-in-Chief for *npj Biofilms and Microbiomes,* I wish this journal to become a platform for cross-disciplinary discussions and scientific exchange that allow for the understanding of the biology and ecology of biofilms, microbial populations and communities, as well as applications so derived across medical, environmental and engineering domains.

